# Cost Minimization Analysis of Hypofractionated Radiotherapy

**DOI:** 10.3390/curroncol28010070

**Published:** 2021-01-30

**Authors:** Hannah L. Yaremko, Gordon E. Locke, Ronald Chow, Michael Lock, Robert Dinniwell, Brian P. Yaremko

**Affiliations:** 1 Department of Basic Medical Sciences, Western University, London, ON N6A 3K7, Canada; hyaremko@uwo.ca; 2 Department of Radiation and Oncology, University of Toronto, Toronto, ON M5S 1A1, Canada; gordon.locke@rmp.uhn.ca; 3 Yale School of Public Health, Yale University, New Haven, CT 06510, USA; ronald.chow@yale.edu; 4 Department of Oncology, London Regional Cancer Centre, Western University, London, ON N6A 4L6, Canada; michael.lock@lhsc.on.ca (M.L.); robert.dinniwell@lhsc.on.ca (R.D.)

**Keywords:** breast cancer, cost minimization analysis, hypofractionation, radiation

## Abstract

Early-stage breast cancer patients comprise a large proportion of patients treated with radiotherapy in Canada. Proponents have suggested that five-fraction hypofractionated radiotherapy for these patients would result in significant cost savings. An assessment of this argument is thus warranted. The FAST-Forward and UK FAST clinical trials each demonstrated that their respective hypofractionated regimens provided equivalent outcomes compared with standard regimens. Thus, a cost-minimization analysis was performed to quantify the potential savings associated with these regimens, which were designated as FAST-Forward 1 (26 Gy/5 fractions/1 week) and FAST-Forward 2 (27 Gy/5 fractions/1 week), and UK FAST 1 (28.5 Gy/5 fractions/5 weeks) and UK FAST 2 (30 Gy/5 fractions/5 weeks). A standard regimen of 42.5 Gy/16 fractions/5 weeks was also included. A comprehensive model of radiotherapy costs for a Canadian cancer centre was created. Time, labour costs, and capital costs were calculated for each regimen and applied using established measures. The total costs per patient for the FAST-Forward trials were $851.77 for FAST-Forward 1 and $874.77 for FAST-Forward 2, providing a total savings of $487.99 and $464.99, respectively. Similarly, the total costs per patient for the FAST trials were $979.75 for UK FAST 1 and $1017.70 for UK FAST 2, providing savings of $360.01 and $322.06, respectively. Following the FAST-Forward 1 regimen results in the greatest reduction of infrastructure and human resources costs at 36.42% compared with the standard. Sensitivity analysis shows a maximum per-patient costs savings ranging from $474.60 to $508.53 for the FAST-Forward 1 trial, which translates to an annual savings of $174,700/year locally and $2.06 million/year province-wide, based on a moderate-to-large size department workload. Compared with a standard radiotherapy regimen, all FAST-Forward and UK FAST hypofractionated regimens provide cost savings for the treatment of early-stage breast cancer. The cost savings associated with each of these equivalent regimens can be directly calculated; activities in this model can easily be adjusted to account for cost variations, allowing other centres to calculate cost impacts specific to their own centres.

## 1. Introduction

The current standard of care for early-stage breast cancer (commonly defined as pT1–3a, pN0–1, M0) is lumpectomy followed by adjuvant radiotherapy to decrease the risk of local recurrence [1]. Although this basic approach has remained unchanged over several decades, the nature of the radiotherapy component itself has experienced considerable evolution. The historic gold standard regimen was 50 Gy in 25 fractions, delivered to the whole breast. Such regimens were devised according to the principle of standard-fractionation, whereby dose is delivered at 1.8-2 Gy per fraction [2]. For the last several decades, there has been increasing use of adjuvant whole-breast radiotherapy using hypofractionation, whereby a clinically equivalent dose is delivered in a smaller number of fractions, typically at >2 Gy per fraction [3]. The safety and effectiveness of hypofractionation has now been proven conclusively via several randomized-controlled clinical trials. For example, the UK Standardisation of Breast Radiotherapy trials (START A and START B) were landmark, large, multi-centre clinical trials that investigated if patients with early breast cancer could be treated safely with hypofractionated whole-breast radiation therapy after lumpectomy. START A compared three different whole-breast treatment regimens, with roughly 750 women randomized to each arm: 50 Gy in 25 fractions, 41.6 Gy in 13 fractions, and 39 Gy in 13 fractions. START B compared 50 Gy in 25 fractions with 40 Gy in 15 fractions over 3 weeks [4]. Similarly, a well-known Canadian randomized-controlled study compared 50 Gy in 25 fractions to 42.5 Gy in 16 fractions [5]. Such studies have established hypofractionated radiotherapy as the new standard for adjuvant, whole-breast radiotherapy.

More recently, two major trials have pushed hypofractionated treatment even further: UK FAST and FAST-Forward [6]. UK FAST was a randomized controlled trial wherein 915 women were randomly assigned to one of three treatment arms: a standard 50 Gy in 25 fractions or two similar 5-fraction regimens, either 5.7 Gy per fraction (28.5 Gy in total) or 6 Gy per fraction (30 Gy in total), with each fraction given 1 week apart, for a total of 5 weeks. Analysis of 10-year outcomes from UK FAST was recently published [6,7]. The cumulative incidence rate of ipsilateral recurrence was 1.3% after 10 years, with a total of 96 patients dying, with 25 of these deaths being cancer-related. Their analysis suggested that the hypofractionated schedule of 28.5 Gy in 5 fractions once weekly was an appropriate alternative to the standard regimen of 50 Gy in 25 fractions. FAST-Forward trial was an even larger multi-centre randomized controlled trial comprising 4096 patients. Women were randomly assigned to one of three regimens: a standard regimen of 40 Gy in 15 fractions over 3 weeks, or hypofractionated regimens of either 5.2 Gy or 5.4 Gy per fraction given once weekly, over 5 weeks, for a total of 26 Gy or 27 Gy, respectively. Each of these regimens was equivalent to the historical standard. At a median of 71.5 months after treatment, 79 patients had experienced ipsilateral relapse; 31 in the standard group, 27 in the 27 Gy group, and 21 in the 26 Gy group. Reported normal tissue toxicities were also acceptable. The five-fraction regimens were found to be non-inferior to the standard 3-week regimen, while the 26 Gy regimen was similar to the standard in terms of breast appearance and normal tissue effects [8].

Such results, therefore, suggest that hypofractionated whole-breast treatment regimens could represent safe, practicable alternatives to traditional conventionally fractionated treatment regimens. If such ultra-brief regimens could be implemented on a large scale, as a new standard-of-care, considerable benefit could ensue for both the patient and the healthcare system. For example, patient throughput is increased, the number of treatment slots is reduced, and resource utilization and overall costs can potentially be decreased [9,10]. Additionally, many cancer patients require time away from work during treatment, or they live far away from the treatment centre and must relocate during treatment. The opportunity to complete treatment over the course of a few days as opposed to many weeks could help alleviate such social and financial pressures, and it could even entice more women to accept treatment who might otherwise refuse [11].

Public health care systems are under considerable financial burden and will likely remain this way over the coming decades. Implementing treatment plans that reduce costs and consume fewer resources could result in significant financial savings to taxpayers, while the opportunity to do so for breast cancer patients, who make up a large portion of all cancer patients treated worldwide, makes such an innovation especially attractive. At the same time, because of the ongoing COVID-19 pandemic, radiation oncologists have been under unprecedented pressure to deliver treatment while reducing potential exposure, something that aligns very nicely with such abbreviated radiation treatment regimens. Similarly, should COVID-19 (or any other future pandemic or exceptional crisis) necessitate that breast cancer surgeries be delayed or cancelled to reduce transmission of the virus, such abbreviated regimens could again prove valuable to manage any treatment backlog that might ensue once the operating rooms are re-opened.

Clearly, such regimens present considerable clinical and fiscal opportunity, but they must be assessed very cautiously before large-scale implementation. Because hypofractionated treatment regimens are becoming increasingly frequent treatment options in Canadian hospitals [12], it is important to have accurate information related to their associated costs or savings. For such reasons, we performed a detailed analysis of these novel regimens from a typical Canadian perspective, comparing a standard radiotherapy treatment regimen of 42.5 Gy in 16 fractions over 5 weeks to the hypofractionated regimens seen in the UK FAST and FAST-Forward trials. With the assumption that all such regimens would be characterized by equivalent survival and toxicities, we applied the well-established cost-minimization methodology to determine the fiscal benefits provided by such regimens, to determine if such 5-week or 1-week regimens provide significant cost savings compared with the standard regimen. Based upon analyses such as this, such regimens could potentially become the basis of a new standard-of-care, delivering safe and economical whole-breast radiotherapy to women with early-stage breast cancer, thus improving survival and alleviating financial strain.

## 2. Materials and Methods

### 2.1. Cost Minimization Analysis

Cost-minimization identifies the lowest cost of treatments that have been shown to be otherwise equivalent in clinical effectiveness. It was previously used to demonstrate cost savings in delivering radiation therapy [13]. We used a cost-minimization analysis method to evaluate the costs of the hypofractionated regimens from FAST-Forward and UK FAST studies relative to standard hypofractionated whole-breast radiotherapy. Our analysis focused on direct costs incurred by the department and the health care system; costs incurred by the patient were not included.

We examined four hypofractionated radiotherapy regimens used in the FAST clinical trials: (i) 26 Gy/5 fractions/1 week (in this study, referred to as FAST-Forward 1); (ii) 27 Gy/5 fractions over 1 week (FAST-Forward 2); (iii) 28.5 Gy/5 fractions over 5 weeks (UK FAST 1); and (iv) 30 Gy/5 fractions over 5 weeks (UK FAST 2). All of these were compared to our standard regimen, 42.5 Gy/16 fractions over 3½ week. All regimens were assumed to be equally effective in terms of potential toxicities and clinical effectiveness.

For each regimen, a detailed analysis was performed to define the per-patient costs of equipment, human resources, and other logistical resources needed. All values are in Canadian dollars. A process map previously developed by Leung et al. [13] was used to identify all resources and associated time requirements as the patient moved through the various components of treatment planning and delivery. This map included time required for CT simulation, contouring, dosimetry, quality assurance, and treatment. An activity-based methodology [14] was used to determine the costs for each resource, which were then defined via a detailed chart review, interviews with treatment planning and delivery staff, and a detailed literature review. Median values were chosen for time and wages, and sensitivity analyses were performed to determine the impact of maximizing or minimizing costs.

### 2.2. Cost

The direct cost of treatment can be divided into infrastructure and human resource costs. The number of hours needed to deliver a complete radiotherapy treatment was determined for radiation oncologists, radiation physicists, and radiation therapists through literature review and interviews. Timing data for the steps prior to treatment were verified to ensure accuracy using process monitoring methods. In accordance with the Consolidated Health Economic Evaluation Reporting Standards (CHEERS), this paper reports the time horizon and discount rate [15]. The time horizon is one year with a discount rate of zero. The one-year time horizon was chosen as the hospital budget is typically determined for a twelve-month fiscal period. The discount rate of zero was chosen as we do not expect costs to change during this single-year time frame.

### 2.3. Human Resources Costs

The salaries and hourly wages of each profession were obtained from the collective bargaining agreement signed by each profession. Radiation therapy has hourly wages included in its collective bargaining agreement published by the Canadian Association of Medical Radiation Technologists [16]. The Professional Institute of the Public Service of Canada publishes the salary ranges for medical physicists [17]. Human resource activities for each step in the process map were assigned a specific time. These were validated through staff interviews, real-time process monitoring of patient throughput, and review of relevant articles described in Leung et al. [13].

### 2.4. Infrastructure Costs

The costs related to infrastructure needed to deliver the radiation are also included in this analysis. This includes the purchasing of the machinery needed to generate and deliver the radiation. The expected lifespan of a linear accelerator (LINAC) is approximately 10 years according to the manufacturer’s guidelines for replacement. The purchasing price of a new linear accelerator has a range depending on the features and specifications. This study determined the purchasing price of a dual energy, intensity-modulated radiation therapy (IMRT), volumetric modulated arc therapy (VMAT) and image-guided radiation therapy (IGRT)-equipped linear accelerator to be $4 Million. Given the operational lifespan of 10 years, the investment in each year of operation is $400,000 and using the provincial standard of 3.4 fractions an hour, 253 days of operation a year, with 10 h a day of operation, a LINAC can be expected to deliver 8602 fractions of radiation per year. This gives each fraction a cost of ($400,000/year) ÷ (8602 fractions/year) = $46/fraction. Therefore, for a standard 2Gy/fraction, the annual depreciated LINAC cost is ($46/fraction) ÷ (2Gy/fraction) = $23 per Gray.

### 2.5. Excluded Costs

This study does not include costs indirectly associated with the radiation therapy treatment that are outside of the hospital’s operating budget and costs incurred by the patients. Furthermore, indirect costs, including the provision of hospital administration (management, clerks), information technology (the management of patient health records), and the maintenance costs needed to maintain the linear accelerators, were considered fixed costs, common to all regimens, and were not included. Finally, no regimen was assumed to be more onerous than any other in terms of time-demands upon the physician (i.e., time required for contouring, planning, and set-up would be identical with every regimen, as would the time required for consenting, counselling, monitoring, or managing patients on treatment).

## 3. Results

### 3.1. Human Resources Time Requirements

The total number of hours for human resources was calculated for each fractionation regimen; the time required to deliver one fraction was calculated and a slot correction factor was applied to account for variation. The standard regimen required 5.33 h of radiation therapist time. The FAST-Forward 1 and FAST-Forward 2 regimens required 2.71 h, and the UK FAST 1 and UK FAST 2 regimens required 2.58 (Table 1). The medical physicist time and dosimetry time requirements were assumed not to differ for all treatment regimens.

### 3.2. Human Resource and Infrastructure Costs

The applicable human resource hours were multiplied by the hourly rate to provide the direct per-patient human costs for each regimen. Infrastructure costs were determined by multiplying the number of fractions by the $23/Gy annually depreciated LINAC cost for each regimen. Human resource and infrastructure costs were added to determine the total estimated cost per patient (Table 2). The standard regimen results in a per-patient cost of $1339.75. The hypofractionated regimens showed much lower costs in comparison; for example, the FAST-Forward trial predicts a total cost of $851.77, a savings of $487.99 compared with standard (Table 3). The additional cost of unfilled slots was accounted for using a 10% slot correction factor; this was only applicable to the UK FAST regimens as the FAST-Forward regimens occurred over just one week. The cost per patient of unfilled slots was $65.55 for FAST-Forward 1 and $69.00 for FAST-Forward 2.

Compared with the standard 16-fraction regimen, the human resource costs for each of the comparison regimens is substantially less, ranging from 28.6 to 29.95 percent. Based upon average Humane Resources HR costs and average infrastructure costs, none of the hypofractionated regimens are substantially different from one another. However, all hypofractionated regimens are substantially different from the standard regimen in terms of overall cost. Of these hypofractionated regimens, the lowest cost is FAST-Forward 1. The total cost per patient dropped from 1339.75 for the standard regimen down to between $851.77 for FAST-Forward 1 and $1017.70 for UK FAST 2 (Table 3).

Sensitivity analysis was then performed to compare the hypofractionated and standard regimens. The total costs of each treatment are dependent on the hourly wages, treatment times, and infrastructure costs. The costs were compared using the lowest and highest values found through our study’s data collection. The total costs were then calculated to find the minimal and maximal costs for each treatment and to find the smallest and largest projected cost savings. Again, FAST-Forward 1 showed the greatest benefit in general during sensitivity analysis; this benefit persisted on sensitivity analysis regardless of whether variable costs were maximized or minimized. For the maximum cost in which human resource costs are maximized, the savings compared with the standard were $508.53. With all labour costs minimized, the savings compared with standard were $474.60 (Table 4).

## 4. Discussion

There has been a rapid expansion in the use of hypofractionated radiotherapy for low-risk breast cancer. A wide variety of treatment regimens and techniques have been used, including treatment of the whole breast or treatment of the cavity alone, preoperatively or postoperatively, using photons, brachytherapy, or other more specialized treatment delivery platforms. The underlying presumption with all of them is that they will provide an equivalent effect in terms of reducing the risk of local recurrence within the breast itself. Thus, based solely on its ability to produce a desired clinical effect, any of these regimens would be effective options for treating early-stage breast cancer. This presumption also lends itself nicely to performing cost minimization analysis, which itself demands that all entities being compared be characterized by outcomes that can be assumed to be the same.

However, while a particular clinical outcome—such as local recurrence—might be equivalent between two given treatment regimens, such regimens might still differ in some other relevant outcome—such as treatment-related toxicity, especially late effects. Although there is now extensive follow-up available for both UK FAST regimens, as well as for the standard 16-fraction regimen, the FAST-Forward regimens are still very new, and correspondingly less well understood. Therefore, without long-term follow-up and analysis, we cannot know for sure whether these hypofractionated regimens would differ in terms of variables such as toxicity. It is certainly possible that future study will show enhanced toxicity for one of these regimens versus another. For example, consider the RAPID study (15), a multi-centre, randomized trial based in Canada, Australia, and New Zealand, which implemented a hypofractionated regimen of 38.5 Gy in 10 fractions over 5 days using accelerated partial breast irradiation (APBI). The women treated using the experimental hypofractionated regimen had non-inferior clinical outcomes to those treated using the standard control regimens. Both regimens were well tolerated at first (interestingly, patients treated with APBI actually had less acute radiation toxicity 3 months after radiotherapy was given). However, at an 8-year follow-up, women treated with APBI had significantly worse late toxicity, including adverse cosmesis. Similarly, B-39/RTOG0413 randomized patients to APBI or standard whole breast-radiotherapy regimens (16). At a median follow-up of 10 years, APBI was statistically significantly inferior to whole-breast irradiation and had worse cosmetic outcomes (though, overall, the absolute differences between arms were very small).

Although the treatment regimen used in both these studies was clinically acceptable in terms of its ability to reduce recurrence, fear of such treatment-related late toxicity has likely impeded the implementation of APBI in Canadian healthcare centres and suggests that further follow-up and investigation into these late effects is warranted. As an example, the OPAR trials were developed as a successor to RAPID (ClinicalTrials.gov Identifier NCT02637024). In OPAR, two different fractionation schemes were considered to treat the lumpectomy cavity (27 Gy and 30 Gy, each delivered over 5 daily fractions). OPAR gave similar doses to the hypofractionated regimens examined here, but the fractionation was adjusted to address the perception that the toxicity in RAPID was too high and needed to be investigated.

If there is an underlying, thus-far occult risk of enhanced late toxicity associated with any of the five fraction regimens considered here, it could very well render the assumptions incorrect. Although minor, RAPID/RTOG0413 both suggested a potential difference in toxicity compared with the standard approach (15, 16). Given the design of each of the U.K. studies, such a difference in late effects could potentially be amplified further. Specifically, in UK FAST and FAST-Forward, similar doses to RAPID/RTOG0413 were used, but they were given over 5 fractions, not 10, while the whole breast was treated, not just the cavity. On the other hand, such a difference could be mitigated if the standard-fractionation regimen (42.5 Gy in 16 fractions) was actually dosimetrically greater than any of the U.K. studies. Using the standard linear-quadratic formulation (assuming α/β = 10 Gy), 42.5 Gy in 16 fractions is equivalent to a dose of 28.0 Gy in 5 daily fractions for equivalent late effects. This dose is actually slightly greater than either of the FAST-Forward regimens, and arguably substantially more than either of the UK FAST regimens, given the 1 week between treatments, during which a substantial amount of normal tissue recovery (as well as tumor proliferation) could potentially occur. Of course, at such large doses per fraction, the linear quadratic model is itself probably approaching the limits of its utility as a conversion tool [18].

Based strictly on cost minimization, and assuming equivalent clinical efficacy, the FAST-Forward 1 regimen is the most attractive option. However, as our results show, all four of the U.K. regimens that we analyzed cost roughly the same, and all four of them are expected to provide considerable savings relative to the standard 16-fraction approach. As such, it is possible that a given treatment centre might choose to offer both a daily regimen and a weekly regimen, with the expectation that the patient can choose which regimen might work best for her based upon her own individual reasons, such as travel or personal costs and time. In either case, the centre would enjoy a substantial cost savings over the current standard whole-breast approach.

The London Regional Cancer Program delivers radiation to approximately 4000 new patients annually (14). In 2019, patients with previously untreated breast cancer (all stages) accounted for 21% of all patients treated at our centre (678 patients in total). Patients with early-stage breast cancer (stage 0 and stage 1) accounted for approximately half (48%) of these, representing 10% of all patients treated at our centre (323 patients in total). Therefore, adopting the FAST-Forward 1 hypofractionated regimen (26 Gy in 5 fractions over 1 week) for all patients with early (stage 0 and stage 1) breast cancers who would otherwise be eligible for treatment with a standard 16-fraction regimen could generate savings of (358 patients/year) ($487.99/patient) = $174,700/year. Such savings become even more substantial when we extend this out to the entire province of Ontario. In 2019, there were 4226 patients treated with adjuvant radiotherapy for early-stage breast cancer (stage 0 and stage 1) province-wide. In the same manner as above, this amounts to a potential savings of (4226 patients/year) ($487.99/patient) or $2.06 million/year.

In these remarkable times where we are still in the midst of a pandemic, possibly for several more years, any of these hypofractionated regimens is potentially attractive as they can minimize the potential exposure of staff, family members, and patients to the virus. Studies have shown that a small number of fractions might potentially entice more women to come for treatment. These regimens are viable options that could help properly balance patient quality of life and risk of COVID-19 exposure when developing treatment regimens. In this case, cost utility analysis should be performed to assess whether the benefits of these hypofractionated regimens are worth the potential toxicity risks.

## 5. Conclusions

This cost-minimization model has demonstrated that adopting a hypofractionated schedule, such as those in the UK FAST and FAST-Forward trials, is more fiscally responsible, providing significant savings to the healthcare system in addition to potential non-financial benefits to the patients and caregivers. Cost variations in this model can be adjusted for each activity to allow other centres to quickly determine detailed cost impacts specific to their own centres.

## Figures and Tables

**Table 1:** Summary of human resources required in hours per patient per treatment course.

		FAST-Forward 1	FAST-Forward 2	UK FAST 1	UK FAST 2	Standard
		26 Gy in 5 Fraction/1 Week	27 Gy in 5 Fractions/1 Week	28.5 Gy in 5 Fractions/5 Weeks	30 Gy in 5 Fractions/5 Weeks	42.5 Gy in 16 Fractions/5 Weeks
Radiation Therapist						
	CTSim	0.50	0.50	0.50	0.50	0.50
	CTSim Quality Assurance (QA)	0.25	0.25	0.25	0.25	0.25
	Calc Station	0.58	0.58	0.58	0.58	0.58
	Treatment	1.25	1.25	1.25	4.00	4.00
	Unfilled slot correction factor	0.00	0.00	0.13	0.13	N/A
	SUM	2.58	2.58	2.71	2.71	5.33
Medical Physicist						
	Physics QA	0.27	0.27	0.27	0.27	0.27
	Physics	1	1	1	1	1
	SUM	1.27	1.27	1.27	1.27	1.27
Dosimetrist						
	Dosimetry	1.50	1.50	1.50	1.50	1.50
	Dosimetry QA	0.27	0.27	0.27	0.27	0.27
	SUM	1.77	1.77	1.77	1.77	1.77

**Table 2:** Summary of human resource and infrastructure expenditures.

		FAST-Forward 1	FAST-Forward 2	UK FAST 1	UK FAST 2	Standard
		26 Gy in 5 Fraction/1 Week	27 Gy in 5 Fraction/1 Week	28.5 Gy in 5 Fractions/5 Weeks	30 Gy in 5 Fractions/5 Weeks	42.5 Gy in 16 Fractions/5 Weeks
Human Resources	Hourly Rate	Cost per patient (CAD)				
Radiation Therapist	$39.45	101.78	101.78	106.71	106.71	210.27
Medical Physicist	$68.97	87.59	87.59	87.59	87.59	87.59
Dosimetrist	$36.38	64.39	64.39	64.39	64.39	64.39
Total Human Resource Expenditures		253.77	253.77	258.70	258.70	362.25
Linac Expenditures		598.00	621.00	655.50	690.00	977.50
Unfilled Slot Correction Factor		0.00	0.00	65.55	69.00	N/A
Total Infrastructure Expenditures		598.00	621.00	721.05	759.00	977.50

**Table 3:** Total cost per patient associated with standard and hypofractionated radiotherapy.

	FAST-Forward 1	FAST-Forward 2	UK FAST 1	UK FAST 2	Standard
	26 Gy in 5 Fraction/1 Week	27 Gy in 5 Fraction/1 Week	28.5 Gy in 5 Fractions/5 Weeks	30 Gy in 5 Fractions/5 Weeks	42.5 Gy in 16 Fractions/5 Weeks
	Cost per patient (CAD)				
Human Resource Expenditures	253.77	253.77	258.70	258.70	362.25
Infrastructure Expenditures	598.00	621.00	721.05	759.00	977.50
Total Expenditures	851.77	874.77	979.75	1017.70	1339.75
Savings (HR): new regimen compared with standard	108.49	108.49	103.56	103.56	0.00
Savings (Infrastructure): new regimen compared with standard	379.50	356.50	256.45	218.50	0.00
Total savings: new regimen compared with standard	487.99	464.99	360.01	322.06	0.00

**Table 4:** Sensitivity analysis.

	FAST-Forward 1	FAST-Forward 2	UK FAST 1	UK FAST 2	Standard
	26 Gy in 5 Fraction/1 Week	27 Gy in 5 Fraction/1 Week	28.5 Gy in 5 Fractions/5 Weeks	30 Gy in 5 Fractions/5 Weeks	42.5 Gy in 16 Fractions/5 Weeks
All Variable Costs Maximized (CAD)	906.94	958.25	1035.85	1073.80	1415.47
All Variable Costs Minimized (CAD)	828.57	851.57	955.94	993.89	1303.16
Savings—Maximum standard breast radiotherapy minus Maximum hypofractionated regimen (CAD)	508.53	457.22	379.62	341.67	0.00
Savings—Minimum standard breast radiotherapy minus Minimum hypofractionated regimen (CAD)	474.60	451.60	347.22	309.27	0.00

## Data Availability

The data presented in this study are available on request from the corresponding author. The data are not publicly available due to confidentiality.
